# Factors Affecting the Uptake of HIV Testing among Men: A Mixed-Methods Study in Rural Burkina Faso

**DOI:** 10.1371/journal.pone.0130216

**Published:** 2015-07-01

**Authors:** Manuela De Allegri, Isabelle Agier, Justin Tiendrebeogo, Valerie Renée Louis, Maurice Yé, Olaf Mueller, Malabika Sarker

**Affiliations:** 1 Institute of Public Health, Faculty of Medicine, University of Heidelberg, Heidelberg, Germany; 2 University of Montreal School of Public Health (ESPUM), Montreal, Canada, Research Centre of the University of Montreal Hospital Centre (CRCHUM), Montreal, Canada; 3 Centre de Recherche en Santé de Nouna, Nouna, Burkina Faso; 4 James P. Grant School of Public Health, BRAC University, Dhaka, Bangladesh; Duke University Medical Center, UNITED STATES

## Abstract

**Background:**

This study aimed to explore factors shaping the decision to undergo Human Immunodeficiency Virus (HIV) testing among men in rural Burkina Faso.

**Methods:**

The study took place in 2009 in the Nouna Health District and adopted a triangulation mixed methods design. The quantitative component relied on data collected through a structured survey on a representative sample of 1130 households. The qualitative component relied on 38 in-depth interviews, with men purposely selected to represent variation in testing decision, age, and place of residence. A two-part model was conducted, with two distinct outcome variables, i.e. “being offered an HIV test” and “having done an HIV test”. The qualitative data analysis relied on inductive coding conducted by three independent analysts.

**Result:**

Of the 937 men, 357 had been offered an HIV test and 97 had taken the test. Younger age, household wealth, living in a village under demographic surveillance, and knowing that HIV testing is available at primary health facilities were all positively associated with the probability of being offered an HIV test. Household wealth and literacy were found to be positively associated, and distance was found to be negatively associated with the probability of having taken an HIV test. Qualitative findings indicated that the limited uptake of HIV testing was linked to poor knowledge on service availability and to low risk perceptions.

**Conclusion:**

With only 10% of the total sample ever having tested for HIV, our study confirmed that male HIV testing remains unacceptably low in Sub-Saharan Africa. This results from a combination of health system factors, indicating general barriers to access, and motivational factors, such as one’s own knowledge of service availability and risk perceptions. Our findings suggested that using antenatal care and curative services as the exclusive entry points into HIV testing may not be sufficient to reach large portions of the male population. Thus, additional strategies are urgently needed to increase service uptake.

## Introduction

Given increasing anti-retroviral treatment (ART) availability, in 2006, the United Nations called for HIV testing services in resource-poor settings to be scaled up[1]. Knowledge of HIV sero-status is a gateway towards ensuring universal access to HIV/AIDS prevention, treatment, and care. Furthermore, studies have demonstrated the efficacy of HIV testing in decreasing sexual risk behaviours in generally healthy populations[2–4]. However, the literature has consistently reported a major gender gap in participation in HIV testing across Sub-Saharan African (SSA) countries, in spite of increased service availability. Recent national population surveys conducted in low and middle income countries, including SSA, showed that 12% of women and 7% of men report having had an HIV test in the previous twelve months, while34% of women and 17% of men report ever having undergone HIV testing[5–11]. The gender gap in testing has important implications since HIV testing gives the opportunity to engage in care programs[12]andin secondary prevention, particularly so in countries where HIV prevalence is low[7,9]. Men's utilization of HIV testing is essential because as household head,men control decisions and resources that are essential for HIV prevention and care among women[10].

Still, the recent HIV literature on men has focused primarily on ART and their role in the Prevention of Mother to Child Transmission (PMTCT)services[7,9,11,12], but has paid very limited attention to the analysis of male HIV testing patterns. Only a handful of studies, all of which applying exclusively either quantitative or qualitative methods[5,8,10,13–17],has looked into individual and system structures likely to explain what compels men to undergo an HIV test. A study relying on a discrete choice experiment exploring stated preferences for testing among men and women pointed to the importance of heterogeneous gender-specific preferences in testing[18]. In Burkina Faso, the few quantitative surveys on HIV testing status among men are limited either to the HF visitors or to the professionals such as health workers or teachers[11,19,20].

In general, the literature has highlighted a positive association between the likelihood of men having tested for HIV and older age, better HIV knowledge, higher income, higher educational and socio-economic status[17,21–23]. Proper understanding of the complexity of the decision-making process which leads men to undergo an HIV test is still missing.

Our mixed methods,population-based study relied on a combination of quantitative and qualitative methods to explore the complexity of the issue, by both measuring (quantitative) and explaining (qualitative) factors associated with men’s HIV testing behavior in rural Burkina Faso.

## Methods

### Study setting

The study took place in 2009 in the Nouna Health District, north-western Burkina Faso. At the time of the study, the district had a population of approximately 311,000 distributed in 300 villages and included 31 first-line facilities, *Centres de Santé et de Promotion Sociale* (CSPS),and one district hospital, also located in Nouna town. A sub-portion of the district has been part of a Health and Demographic Surveillance System (HDSS) for over 15 years[24].

In line with national estimates[25], adult HIV prevalence in the area has remained stable at 3.6%[26]. At first, HIV counseling and testing based on an “opt-in” strategy was only available in the district hospital (2003–2006). Following national directives[27] starting in 2006, all CSPS were equipped to offer HIV counseling and testing to pregnant women and their partners as the entry point to PMTCT services. Explicit efforts were made to address the need for couple counseling and testing, encouraging spouses to be tested together with their pregnant wives. In addition, providers were encouraged to offer testing to anyone requesting it and/or to anyone who presented with potential symptoms of infection. Findings from a parallel study confirmed the availability of test kits even in the rural facilities[28]. The service was structured to ensure that blood samples could be drawn in the rural facilities and then regularly transported to the district hospital for laboratory testing. Both pre-test and post-test counseling were scheduled to take place at the facility where the blood sample was taken.

### Study design

This population-based study adopted a triangulation mixed methods design[29]. The triangulation design was chosen as the most appropriate mixed methods design, since the research question focused on one single phenomenon, i.e. the decision to test or not to test for HIV, but aimed at capturing all its possible dimensions, in such a way that neither qualitative nor quantitative methods alone would have been able to do. The conceptual framework guiding the study recognized the decision to test or not to test for HIV as the product of the interplay between access factors (acting at the individual and at the community level) and individual opinions, beliefs, and practices related to knowledge, risk perceptions, and perceived vulnerability. The quantitative component relied on household survey data to identify socio-demographic, economic, and health system factors associated with access toHIV testing. The qualitative component relied on in-depth interviews to explore men’s motivation to test or not to test in relation to their knowledge, attitudes, and perceived vulnerability to the disease.

### Study population, sampling, and data collection

#### Quantitative Component

The sample for the quantitative component was defined by the total number of male household heads interviewed in the 2009 round of the panel survey in which we embedded our module on male HIV testing. In line with the overall sampling strategy applied to the household survey, only men aged 20 and above were interviewed as household heads. Details of the household selection procedures have been described in detail in earlier manuscripts which relied on previous rounds of the panel survey to answer other research questions[30–32]. In brief, first, clusters were defined according to the catchment area of each CSPS. Second, two villages within each cluster were selected. Third, 20 households were randomly selected in each village (70 in Nouna town), using modified EPI sampling procedures[33], resulting in a total of 1130 households. Considering that 52 out of 1110 household heads who participated in the 2009 survey round were women, the total sample for the quantitative component amounted to 1058 men.

Data for the quantitative component was collected between February and April 2009 using a close-ended structured questionnaire, which included questions on the overall household socio-economic and health seeking behavior profile. The *ad hoc* module, which exclusively targeted male household heads, collected information on the participants’ knowledge of the availability of HIV/AIDS services in the area, offer to undergo an HIV test (i.e. defined as being offered the opportunity to do a test), and actual behavior concerning testing.

Trained interviewers fromthe *Centre de Recherche en Santé de Nouna*(CRSN) carried out the interviews under the authors’supervision. Independent double data entry took place in Nouna, using Access (Microsoft ACCESS, version 97).

#### Qualitative study component

The sample for the qualitative component was purposely selected on the basis of very preliminary data emerging from the quantitative component. This explains the small delay in data collection (about three months) between the quantitative and qualitative phases. Thirty-eight men were identified from the household survey records, 19 who had tested and 19 who had not tested. In addition to testing status, as purposive selection criteria, qualitative sampling took into consideration geographical distribution (men were selected from 18 villages spread across the entire District) and age (ranging from 30–60 years). The age range for the qualitative sample differs from that of the quantitative component given that survey data revealed that only 30 out of a sample of over 1000 household heads were younger than 30, indicating a practical impossibility to use younger age as a purposive sampling criteria. Data for the qualitative component was collected between June and August 2009Four interviewers trained to conduct open-ended semi-structured interviews. The interview guide addressed knowledge of HIV infection and available services, perceptions of risk and vulnerability to the disease, and explored men’s motivation to test or not to test. The men to be interviewed were contacted directly by the interviewers and asked whether, being part of the panel survey, they were willing to engage in a follow up, in-depth interview. Interviews lasted between 30 and 60 min, were conducted in a confidential setting, tape-recorded, *verbatim* transcribed and translated into French.

#### Analytical approach

Household survey data were analysed using Stata 11 (Stata Corporation, Texas, USA). The outcome variable was defined as “having tested for HIV”. Since HIV testing could be observed only among those men who had in the first place been offered an HIV test, we initially intended to rely on a Heckman estimation model to take into account the selection bias induced by the need to be offered a test before being able to undergo one[34,35]. Our definition of being offered a test included individuals who voluntarily sought a test/asked to be tested, since technically, one always needsto be offereda test before being able to undergo it.

Since it was impossible to identify an adequate instrumental variable for the efficient computation of a Heckman model, wereverted to applying a two-part model[36]. First, we computed a model to estimate factors associated with the probability of being offered an HIV test. Second, we computed a model to estimate factors associated with the probability of having undergone an HIV test. Model 1 (being offereda test) was computed using the complete sample of men having provided complete answers to the module on HIV testing (N = 937). Model 2 (HIV test behaviour) was computed on the truncated sub-sample of men reporting that they had been offered an HIV test in the first place (N = 357). This analytical approach allowed us to discern factors associated with the probability of being offered a test from those associated with the probability of actually undergoing the test, in order to depict decision-making more realistically. In both instances, we did not aim at saturating the model by exploring all possible factors associated with either level. We rather aimed at identifying a few theoretically relevant factors which could be properly identified in our dataset. Probit estimation accounting for clustering at the village level was used to compute both models. The two equations estimated separately are written as follows:

Model 1: P(offered_i_ = 1/x_1i_) = ф(x_1i_β_1_).
Model 2: P(tested_i_ = 1/x_2i_, offered_i_ = 1) = ф(x_2i_β_2_)

with

offered_i_ = 1 if being offered an HIV test and 0 otherwise.

tested_i_ = 1 if having undergone an HIV test having been offered one and 0 if not (having been offered one).

and ф(.) the normal cumulative distribution function.

x_1i_andβ_1_ are respectively the factors associated with the probability of being offered a test and the vector of coefficients associated with these factors.

x_2i_andβ_2_ are respectively the factors associated with the probability of actually undergoing the test and the vector of coefficients associated with these factors.

Prior to estimating both models, we carried out descriptive analysis to identify bivariate associations between the explanatory variables included in our analysis and the two distinct outcome variables.

Table 1 displays the explanatory variables included in the analysis as well as the expected sign of the coefficient, indicating the *a priori* underlying hypothesis we held. Most variables are straightforward. The decision to dichotomise ethnicity into “Bwaba” and “other ethnicity” was informed by previous research showing that the Bwaba often display different health seeking patterns [37–39]. Standard procedures applying principal component analysis were used to estimate household wealth [40,41].

**Table 1:** Variables and their measurement.

Variables	Measurement	Expected sign in Model 1	Expected sign in Model 1
HIV test offered	0 = no HIV test offered	-	-
	1 = HIV test offered		
HIV test	0 = man has not taken an HIV test	-	-
	1 = man has taken an HIV test		
Ethnicity	0 = not Bwaba	+	+
	1 = Bwaba		
Education	0 = never attended formal school	+	+
	1 = attended formal school		
Marital status	0 = other (single, widow)	-	-
	1 = married (monogamous and polygamous)		
Pregnant partner	0 = partner has not been pregnant in past two years	+	+
	1 = partner has been pregnant in past two years		
Age	Continuous variable (years)	-	-
Household wealth	Continuous index categorized as 1 = lowest quintile (poorest) and 5 = highest quintile (least poor)	+	+
Distance to closest first line facility	Continuous variable (km)	-	-
HDSS	0 = man does not live in a HDSS village	+	+
	1 = man lives in a HDSS village		
Knowledge of HIV service	0 = man does not know that the local facility offers HIV services	+	+
availability	1 = man knows that the local facility offers HIV services		

Analysis of the qualitative material took place on the transcribed material, directly in French. We translated into English only the citations included in this manuscript. First, the materialwas independently coded by three of the authors, all relying on an inductive approach which let codes emerge as the reading proceeded. Second, the three analystscompared their analyses and returned to the material to resolve divergent interpretations. Third, the collective emerging interpretation was discussed among all authors, informed in the light of the quantitative findings, and finalised.

The interpretation of the findings as presented in this paper is based on the joint appraisal of the quantitative and qualitative findings. The process of bringing together in one single conceptual model findings from the two components was conducted at the end of the two distinct analytical approaches.

#### Ethical considerations

Institutional ethical review of the study protocol was obtained from the Ethical Committee of the Faculty of Medicine of the University of Heidelberg, Germany and from the Ethical Board of the CRSN,Nouna, Burkina Faso. Oral consent was obtained from all study participants separately for the quantitative survey and the qualitative interviews. Given very high illiteracy rates, the consent form was read aloud to the potential respondent and his permission obtained before starting the interview. In addition,verbal consent was obtained from the village chief before introducing the survey in a given community. The consent form was approved by both the Heidelberg and the Nouna Ethics Committees.

## Results

To simplify reading and highlight the contribution of the single study components, quantitative and qualitative findings are presented separately in this section before being discussed together later on.

### Quantitative findings

Complete information could be ascertained for 937 (89%) of 1058 male household heads. Selection bias at this level was excluded since no systematic differences were observed between households heads with complete vs. household heads with incomplete information (data not shown). Of the 937 men included in the final sample, 357 (38%) had been offered an HIV test. Bivariate analysis indicated the existence of important differences between the men who had been offered an HIV test before, and the men who had never been offered an HIV test, suggesting the existence of selection bias at this level (Table 2). Men who were offered testing were more likely to be younger, of Bwaba ethnicity, wealthier, live closer to a CSPS and in a HDSS village,tohave attended some form of formal schooling, and to be aware that HIV testing could be performed in a CSPS.

**Table 2:** Descriptive statistics and t-test comparing men being offered and men not being offered HIV test (see Table 1 for variable values).

	Not offered HIV test			Offered HIV test			Pearson or t-test	
	N	Mean	Std dev	N	Mean	Std dev	chi2(1) or Student t	p-value
Ethnicity(*)	581	0.423	0.495	357	0.529	0.500	9.9916	0.002
Education(*)	581	0.172	0.378	357	0.291	0.455	18.4616	0.000
Marital status(*)	581	0.308	0.462	357	0.308	0.462	0.0000	0.999
Pregnant partner(*)	570	0.388	0.488	349	0.441	0.497	2.5690	0.109
Age	580	51.210	15.940	357	46.510	13.611	4.805	0.000
Household wealth Index	580	-0.082	0.914	357	0.166	1.087	-3.586	0.000
Distance to closest first line facility	581	4.281	5.079	357	3.966	5.682	0.856	0.393
HDSS(*)	581	0.299	0.458	357	0.490	0.501	34.4242	0.000
Knowledge of HIV service availability(*)	580	0.747	0.435	357	0.902	0.298	34.1012	0.000

(*) Dummy variable—Preson Chi2 test applies (T-test applies otherwise)

Of 357 men who were offered HIV testing, 97 (27% of the sub-sample being offered testing and 10.3% of the total sample of men included in this study) had taken up the opportunity at any prior point in time. Bivariate analysis revealed that only two factors were significantly positively associated with the probability of having tested for HIV: having attended some form of formal schooling and living closer to a CSPS. Household wealth also showed indication of a positive association with HIV testing (Table 3). Table 4 reports the estimation of the two probit models, one estimating the probability of being offered an HIV test (n = 916) and one estimating the probability of actually having undergone an HIV test once offered it (n = 349). Due to missing values on some explanatory variables, the number of observations included in the estimation of the two models is slightly lower than the total samples indicated above. Younger age, household wealth, living in an HDSS village, and knowing that HIV testing is available at the CSPS were all factors positively associated with the probability of being offered an HIV test (model 1). Household wealth and literacy were also found to be positively associated with the probability of actually having taken an HIV test, while the distance to the closest CSPS was found to be negatively associated with this probability (model 2).

**Table 3:** Descriptive statistics and t-test comparing men who tested and men who did not test for HIV (see table 1 for variable values).

	Not tested for HIV			Tested for HIV			Pearson or t-test	
	N	Mean	Std dev	N	Mean	Std dev	chi2(1) or Student t	p-value
Ethnicity(*)	260	0.504	0.501	97	0.598	0.493	2.5104	0.113
Education(*)	260	0.254	0.436	97	0.392	0.491	6.5077	0.011
Marital status(*)	260	0.304	0.461	97	0.320	0.469	0.0821	0.774
Pregnant partner(*)	254	0.457	0.499	95	0.400	0.492	0.9013	0.342
Age	260	46.612	13.575	97	46.237	13.775	0.229	0.819
Household wealth	260	0.068	1.020	97	0.427	1.216	-2.586	0.011
Distance to closest first line facility	260	4.685	5.882	97	2.041	4.607	4.456	0.000
HDSS(*)	260	0.469	0.500	97	0.546	0.500	1.6831	0.195
Knowledge of HIV service availability(*)	260	0.900	0.301	97	0.907	0.292	0.838	0.837

(*) Dummy variable—Preson Chi2 test applies (T-test applies otherwise)

**Table 4:** Probit regressions of being offered HIV test and being tested (when offered).

	(1)	(2)
	Offered	Tested
VARIABLES^1^	(N = 916)	(N = 349)
***Ethnicity***: Bwaba	0.0734	0.0660
(not Bwaba)	(0.0583)^2^	(0.0699)
***Education***: Attended formal school	0.0731	0.0477
(never attended)	(0.0503)	(0.0610)
***Marital status*:** Married	0.0274	0.0502
(other)	(0.0512)	(0.0656)
***Pregnant partner***: yes, in the past two years	-0.0113	-0.0475
(not in the past two years)	(0.0395)	(0.0526)
Age (in years)	-0.00459***	-0.00306
	(0.00131)	(0.00195)
Household wealth	0.0797***	0.0625**
	(0.0218)	(0.0258)
Distance to closest first line facility (in km)	0.00226	-0.0152*
	(0.00572)	(0.00797)
HDSS village	0.177**	0.0661
(non HDSS village)	(0.0703)	(0.0706)
***Knowledge of HIV service availability*:** Man knows	0.199***	0.0166
(man does not know about it)	(0.0378)	(0.0878)
Wald	85.45	41.96
p-value	0	3.35e-06

^1^Reference modality in parenthesis

^2^ Robust Standard errors adjusted for village clusters

* Significant at the 10% level;

** Significant at the 5% level;

*** Significant at the 1% level.

### Qualitative findings

One of the 38 men selected for the qualitative study component refused to be interviewed. When asked to reveal whether they had ever undergone an HIV test, only eight (and not the expected 19) confirmed the information shared during the quantitative phase of the study, meaning that twenty-eight out of thirty-seven men reported never having tested for HIV.

Knowledge on HIV transmission was generally very high among all respondents, with no striking differences between those who had tested and those who had not tested. Men were aware that HIV could be transmitted both sexually and during pregnancy and were generally also aware that treatment was available both to avoid vertical transmission and to delay progression towards the disease. Very few men indicated the sharing of eating utensils and mosquito bites as potential modes of HIV transmission.

*If you see a woman and you do not know that she is ill and you have sex with her*, *you can get the disease*. *This is why*, *even if you love women*, *you need to protect yourself before having an intimate relationship with them*. *Otherwise*, *it can be severe if you just say that you love them and forget to wear the condom that retains the germs (ID 1250008)*.

Thirty-four out of thirty-seven respondents were aware that a test is available to detect HIV, but only one out of eight men who had tested was aware that the test could be performed at the CSPS. All other men believed that testing facilities were only available in the district hospital in Nouna or at the regional hospital in Dedougou and declared having been tested at one of these two locations. A few respondents mentioned that health workers or volunteers from local NGOs conduct outreach campaigns to promote testing, but that they do not offer testing opportunities during these campaigns.

*It is just in Nouna that you can do the HIV test (ID 106 18 00)*.

Perceived health status was the key motivating factor in deciding whether to undergo or not to undergo the test, both among men who tested as well as among men who did not test. On the one hand, men who had not tested justified their decision not to test with the fact that they felt healthy and therefore perceived no need to undergo the test. They supported their decision not to test with the belief that the illness would have surely manifested itself if they had been infected with HIV. On the other hand, men who had tested explained that upon falling ill, they had feared being infected with HIV and therefore had decided to undergo the test. Only two out of the eight men who reported testing had done so to respond to the request of their pregnant wives, following the couples counselling delivered during antenatal care.

*If I was ill*, *I would accept doing the test to know exactly what I have*. *But I feel well*, *so I do not accept doing the test (ID 150 00 06)*.

*I did the test because I wanted to know what I was ill*. *I did a first test at the CSPS*, *a second one at the hospital in Nouna*, *and then one more at the hospital in Dédougou*, *but they all turned out negative*. *This is what I did not understand*. *I am still looking for the cause of my illness (ID 129 11 00)*.

Men who had not tested generally perceived themselves to be at very low risk of contracting the disease. This low risk perception was motivated by the general low prevalence in the community and by the health status and behaviour of their sexual partners. Similarly to what they argued for their own health status, men also believed that had their sexual partners being infected with HIV, the illness would have manifested itself. Likewise, most men did not expect their wives to be at any risk of having contracted HIV since they trusted them to be faithful.

*It is because of the disease is not very common here*. *Lately*, *we have not seen many cases*. *Some years ago*, *there were many cases*. *But lately*, *we have the feeling that the diseases had regressed*. *(ID 106 01 00)*

*If you know that you do not engage in risky behaviours and your wife is serious*, *then there is no need to test*. (ID 153 06 00)

Views on health workers differed substantially between men who had tested and men who had not tested. Men who had tested reported proximity to the health facility and a general wish to comply with health workers’ sensitisation activities as important factors in motivating their decision to test. On the contrary, men who had not tested often expressed subtle mistrust in the health workers’ ability to keep the test result anonymous. They feared stigma should the result of the test or even the fact that they had tested ever be leaked to the community. In addition, men who had not tested did not trust that the test was offered free of charge and were convinced that they would have had to pay something for it.

*In our village*, *if it is known that you have AIDS*, *you die within ten days*. (ID 134 01 00)

*Nobody told me that the test needs to be paid for*, *but I know that at the level of the health facilities*, *one always needs to pay something*. *I have never heard of any service being delivered for free*. (ID 153 01 00)

## Discussion

This mixed methods study represents a unique contribution to the literature on male HIV testing in two ways. First, the application of mixed methods allowed us to explore a wider set of issues relevant to understanding the decision to test or not to test for HIV. In line with the triangulation design, we did not use the qualitative interviews to explain the quantitative findings, but rather to explore issues that could not be assessed, at least not comprehensively, through the structured questionnaire. At the same time, unlike prior research conducted in Burkina Faso[11,15], using population-based rather than facility-based data allowed us to reach both service users and non-users, providing more realistic estimates of HIV testing in the general male population. Second, the analytical model adopted for the quantitative component was chosen to distinguish the offer to test from the actual decision to test. This analytical approach clearly highlights how having tested for HIV is first the result of system factors affecting overall availability and access to HIV services, and second of individual traits shaping the actual decision to undergo the test. Earlier studies were either exclusively quantitative or qualitative and/or did not clearly distinguish the offer to test from the actual decision to undergo the test[5,10,11,13,15,17,21,23].

Results from our household survey indicated that only 10% of men included in the sample reported ever being tested for HIV, in a region where a previous study reported 40% of women attending antenatal care to have tested[42]. Our finding is aligned with lower bound estimates reported by prior studies in SSA, including Burkina Faso, where rates of HIV testing among men have been observed to range from 11% to 24%[5,8,11,15]. Interestingly, nearly one third (27%) of men being offered a test in our sample had actually taken up the opportunity to be tested. This suggests that barriers to HIV testing first operate at the system level, indicating supply-side features, and then at the individual level, indicating demand-side features. It is plausible to assume that an increase in male HIV testing could be observed if a larger proportion of men were explicitly exposed to the opportunity to do so. Building on existing literature, one can postulate that the offer to test would more likely to be taken up frequently if services reflected heterogeneous preferences in testing between men and women[12,18].

Notwithstanding the low HIV prevalence rates of the region, the limited male participation in HIV testing observed in our study is worrisome. HIV testing represents the single entry point into further HIV prevention and care. HIV positive men who are unaware of their status may continue to engage in unsafe behaviorand may not receive the treatment they are entitled to for free until severely ill, thus ultimately contributing to higher HIV mortality[12,43]. In addition, poor male participation in HIV testing puts women at higher risk of contracting the disease and may discourage them from engaging in testing and in related HIV prevention programs, such as PMTCT[3,43].

In our multivariate analysis, four factors were found to be associated with a higher probability of being offered the test: younger age, higher socio-economic status, residence in the HDSS area, and knowing that the test is offered at the CSPS level. The nature of our study does not allow us to distinguish whether the higher probability to be offered a test for young, least poor men residing in the HDSS area and knowing that the test is available at the CSPS is a demand (i.e. user) or a supply (i.e. provider) driven factor. The most plausible explanation is that these fourfactors are an indication of overall better access to health care services and to information on health and illness. As highlighted in prior studies in the study area [44,45] and elsewhere in Africa[5,8,9], younger, least poor men use health services more. As such, these men are more likely to come in frequent contact with the health care system and therefore enjoy increased chances to hear about HIV and to know that a test is available to diagnose it. Reversely, increased use of health care services also increases one’s chances of being offered the test through frequent encounters with providers. Similarly, in line with prior evidence[46][32], through exposure to the repeated studies that have taken place in the HDSS area[24,26,47,48], it is plausible to assume that both providers and communities in this area have better knowledge of both HIV and HIV services than people residing outside the HDSS area. On the onehand, this is likely to induce providers to offer the test more frequently. On the otherhand, this is likely to induce users to be more aware of HIV and better informed about the availability and benefit of a diagnostic test. The beneficial effect of living in the HDSS area on health seeking has been reported in two prior studies, butfurther qualitative inquiry is needed to understand the mechanismsthrough which this effect is produced.

Our qualitative findings did not indicate that knowledge of HIV transmission modes wasdifferent between men residing within and beyond the HDSS area. Relatively high knowledge on HIV transmission was observed across the study region, suggesting the success of sensitization efforts. However, large disparities were observed in relation to knowledge on the availability of the test in first level facilitiesand toconfidence in the fact that the test wasoffered for free. In addition, concerns were raised on the confidentiality of the test results. These disparities in knowledge on service availability and confidentiality concerns echo findings from other settings[16]and are well aligned with the quantitative findings described in the paragraph above. Theycall into question the need to expand sensitization campaigns beyond the scope of providing information on the disease alone to reinforce service availability and confidentialityin the respect for heterogeneous preferencesOn the truncated sample of men being offered the test, multivariate analysis could only identify two positively associated factors, age and household wealth, and one negatively associated factor, distance, with the probability of taking up the HIV test. These findings arealigned with the existing literature, suggesting that younger age and higher socio-economic status are indications of an increased awareness of the benefit of testing and therefore of a higher propensity to take the test[8,15,17]. In addition, wealthier men are less likely to fear financial barriers and therefore more likely to accept the test, once offered, on the assumption that if needed, they can pay for it. This is likely to be the case in a context where people are sceptical that the test is really offered for free, as clearly shown by our qualitative findings. Considering that distance was found to be negatively associated with the decision to take the test, but not with the probability of being offered the test, this suggests that distance cannot be interpreted as a health system factor acting as a barrier to access, as indicated in other settings[16,18]. It is more plausible that in our context, distance is again an indication of knowledge, as people living away from the facility are less likely to be aware of the availability of the test at the CSPS and/or to believe that it is available for free.

In line with prior research[43], the central role of risk perceptions in shaping the decision to take the test was clearly highlighted by our qualitative findings, which indicated how men living in communities with few known cases of HIV infection do not perceive themselves at risk and therefore see no point in taking the test. This low risk perception is understandable in a context of low prevalence, but at the same time, it is worrisome. It suggests that communities do not adequately estimate the risk of contracting the disease. Interventions are needed to increase awareness about the importance of testing even in a context of low prevalence.

Moreover, our qualitative findings reinforced the widely reported observation [6,7,9,21,23,42,43] that across sub-Saharan Africa men are not sufficiently involved in PMTCT programmes. It is revealing that although all married, only two of the men interviewed reported testing in conjunction with their pregnant spouses. Similarly, respondents rarely mentioned the availability of testing during ANC and PMTCT services, showing little awareness of two of the most important entry points into HIV services. Poor participation of men in HIV testing has been shown to bear a negative impact on women, as their. lack of participation in PMTCT programs has been described as a key reason causing women to drop out of such programs[23,42,49,50].

### Methodological considerations

Notwithstanding the strengths of this study, rooted primarily in its mixed-methods population-based approach, we need to acknowledge a few limitations. First of all, the fact of having interviewed only household heads might have limited the generalizability of the findings. Household heads represent a specific group of men, normally enjoying better access to health care services than the general population [51][52]. Thus, it is not to be excluded that they are more likely than other men to be offered testing, given their more frequent contact with health care services. However, household heads tend to be older than the general male population (average age in our quantitative sample was 46 years) and as such they may perceive themselves at lower risk of contracting HIV than their younger counterparts[5]. It is plausible to assume that the HIV testing rate would have been higher than what we observed in our sample had we been able to conduct the study on a sample of men not restricted to household heads. Given the absence of comparable population-based data from prior studies in Burkina Faso, however, we cannot assess if and to what extent the choice to interview exclusively household heads biased the direction of our estimates.

Only eight out of sixteen men who had originally been identified as having tested during the quantitative survey confirmed having done so during the qualitative interviews. This could be an indication of two alternative circumstances: either men provided socially acceptable answers during the household survey (reporting to have tested although this was not the case), ormen denied being tested during the qualitative interview, out of fear of stigma. We have no way to ascertain with certainty whether the former or the latter case applies to our data. We are, however, more inclined to believe that the latter case applies, since the survey question on testing was accompanied by a number of follow-up questions (location of testing, occasion for testing, etc.), which should have allowed the interviewer to recognize if a respondent was reporting non-realistic information. Qualitative interviews are inevitably more intimate and somewhat more threatening than quantitative surveys, which collect information in an anonymous manner. This imbalance in the qualitative sample represents an obvious weakness in our study design, since we ended up interviewing mostly men who had not(or declared not to have) tested for HIV, while our initial wish was to conduct in-depth interviews with an equal number of men having tested and not having tested. This limited our ability to fully explore what can motivate men to test and to build relevant policy guidance for future sensitization campaigns on the basis of these results.

We also need to acknowledge the limitation of estimating factors associated with the offer to test and with the decision to test using a two-part model rather than a Heckman selection model[34]. Our choice was dictated by the contingency of the dataset. Still, estimating the second equation on the truncated sample of men being offered the test is methodologically inferior to estimating the two equations simultaneously since it does not accurately account for the potential for self-selection into the sample. Lastly, the small pseudo-R2 on both models suggests that the explanatory variables included in the models were only able to explain a limited portion of the variation in the outcome variable. However, the variables included represent the complete range of theoretically relevant variables available in the dataset. We purposely chose not to improve the fit of the model by simply including non-theoretically relevant variables.

## Conclusion

In line with existing literature, our mixed methods study provides evidence that HIV testing among males remains unacceptably low, in spite of the wide availability of testing opportunities through PMTCT programs. Our findings indicate that low uptake of the available services results from a mixture of health system factors, indicating general barriers to access, and motivational factors, such as one’s own knowledge of the disease and perception of risk. Our findings indicate that using ANC and curative services at the exclusive entry points into HIV testing may not be sufficient to reach large portions of the male population, especially in low prevalence regions. Notwithstanding the need to strengthen couple counseling during pregnancy and encouraging male participation in PMTCT programs, additional strategies, such as home based HIV testing or mobile testing units, may prove tobe valuablein increasing the uptake of the service among rural SSA communities.
